# Fracture resistance of endodontically treated maxillary incisors restored with single or bundled glass fiber-reinforced composite resin posts

**DOI:** 10.4317/jced.59373

**Published:** 2022-04-01

**Authors:** Bahram Ranjkesh, Yasser Haddadi, Christian-Aalund Krogsgaard, Andreas Schurmann, Golnosh Bahrami

**Affiliations:** 1 Section for Prosthetic Dentistry, Department of Dentistry and Oral Health, Aarhus University, Aarhus, Denmark; 2 Private practice, Aarhus, Denmark

## Abstract

**Background:**

To compare the fracture resistance of endodontically treated maxillary incisors restored with single versus bundled glass fiber-reinforced composite resin posts (FRC).

**Material and Methods:**

Twenty-four maxillary incisors underwent root canal preparation (1.5-mm-diameter post spaces after canal obturation). Teeth were randomly divided into two groups (n = 12). Two different FRC groups were used for the intracanal post treatment. Single FRC (Rebilda Post system, 1.5 mm diameter) and bundled FRC (Rebilda Post GT, 12 fiber bundles, 1.4 mm diameter) were bonded to the prepared canals using dual-cure resin-based luting cement. Specimens were kept in humid at 37°C for one day. The fracture resistance testing was performed using universal testing machine by applying a compressive static load at a 135° angel to the axis of the teeth. The failure type after fracture was examined by visual inspection.

**Results:**

The fracture resistance of teeth with single FRC (Rebilda Post) and bundled FRC (Rebilda Post GT) were 787 ± 156 and 850 ± 166 Newton, respectively. There was no statistical significant difference between the two groups. Root fracture was the major failure type in both groups.

**Conclusions:**

The fracture resistance of endodontically treated maxillary incisors with bundled FRC (Rebilda Post GT) did not differ from incisors with single FRC (Rebilda Post).

** Key words:**Endodontically-treated teeth, fracture resistance, glass fiber post, intracanal post.

## Introduction

Endodontic treatment is known to increase the risk of biomechanical failure when compared to a vital tooth (1). Extensive destruction of the tooth due to caries or trauma often necessitates obtaining mechanical retention for the crown reconstruction by insertion of intracanal posts (2). However, post space preparation inevitably results in the removal of radicular dentin that may eventually weaken the remaining tooth structure (3). Consequently, the teeth restored with intracanal posts would be more prone to failure in form of root fracture, which stands as one of the major causes of tooth loss (4). Intracanal post with a high elastic modulus may increase the stress distribution at the dentin-post interface, which increases the risk of root fracture (5,6). Numerous factors such as post material (7), residual tooth substance (8), ferrule design and height (9,10), post-core materials (11) influence the fracture resistance of the restored tooth.

Intracanal post systems are available in various materials, forms, and surface textures. Glass fiber-reinforced composite resin (FRC) intracanal posts are commonly used that provide improved esthetic, comparable elastic modulus to dentin , bonding to the tooth structure, which would eventually cause better stress distribution to the remaining dental structure and along the cementation interface (12,13). However, debonding of the fiber posts, and difficulty in adaptation to various root anatomies, e.g. curved root canals and pronounced conicity, stand as one of the limitations of single post fiber-reinforced composite resin posts (FRC). Moisture control, stress induced by cement polymerization inside the root canal, and convenient visualization affect the bonding procedures of FRCs (14).

Recently, bundled FRC (Rebilda Post GT, Voco, Cuxhaven, Germany) has been introduced, which consists of multiple diametrically smaller fiber posts designed to offer enhanced physical properties, better adaption to the root canal morphology, and more conservative post space preparation. The aim of this study was to evaluate the effect of new bundled FRC versus single FRC on the fracture resistance of endodontically treated single canal premolars. The null hypothesis was that there was no difference in the fracture resistance and failure mode of endodontically treated maxillary incisors restored with bundled or single FRC.

## Material and Methods

Caries-free extracted single canal maxillary central and lateral incisors kept in 0.4% thymol were used in the study. There was no need for ethical committee approval since the specimens were anonymous biological materials (i.e. the teeth had already been removed for dental treatment purposes). Crowns were cut 1 mm above the cementoenamel junction with a diamond disc under water coolant. Twenty-four teeth with circular canal form, straight axis, canal patency, and no visual fracture (under 10× magnification) were included in the study. The methodology and sample number was adopted by Hai Qing *et al*. study (15). The teeth were root canal treated. Canal instrumentation was performed using canal preparation rotary system sequence (ProTaper Next, Dentsply Maillefer, Ballaigues, Switzerland) up to size X2 to the working length. The canals were irrigated with 2.5% NaOCl solution between instrumentation and 17% EDTA as the final irrigation to remove the smear layer. The canals were obturated with corresponding single cone gutta-percha point (ProTaper Next, X2 gutta-percha, Dentsply Maillefer, Ballaigues, Switzerland) and root canal sealer (AH Plus, Dentsply Sirona, Ballaigues, Switzerland). The teeth were kept in a moist environment in an incubator at 37°C to allow the proper setting of the sealer.

**Figure 1:**
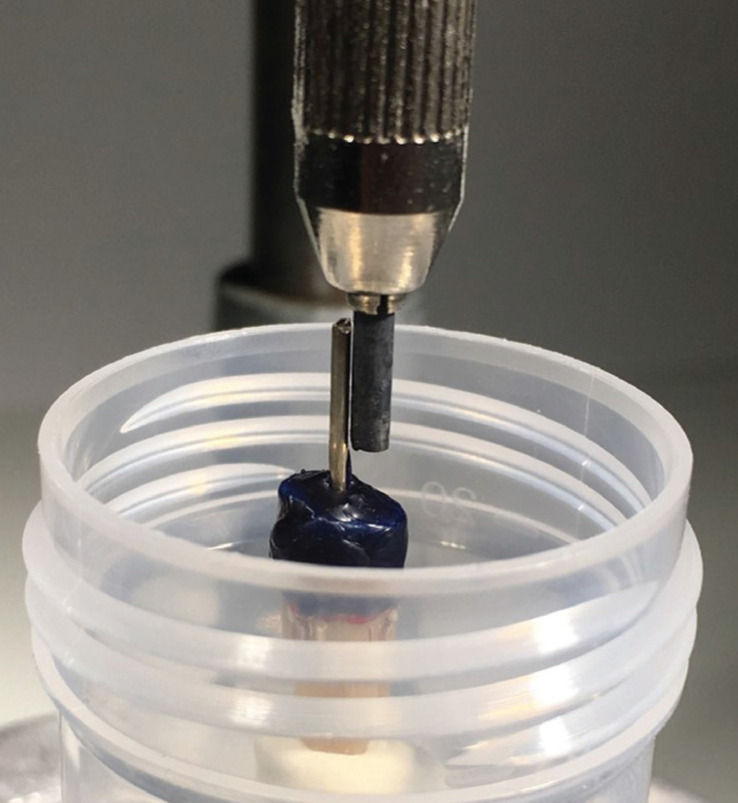
Perpendicular positioning of the specimen using dental cast surveyor before embedding the teeth inside the mold.

After one week, 5-6 mm gutta-percha was initially removed from the canal orifice using Gates-Glidden bur #1 (0.5 mm diameter) to #4 (1.1 mm diameter) (Komet Dental, Lemgo, Germany). A stainless-steel bar (1-mm diameter) was inserted in the canal and temporally secured with wax (Blue Inlay Casting Wax, Kerr, Brea, CA) at the orifice of the canal and almost 3 mm circumferentially around the root. This allowed for properly adjusting the perpendicular position of the specimen using a dental cast surveyor before embedding inside the mold (Fig. 1) and prevention of embedding at the utmost 3-mm-coronal part of the root. A cold-setting embedding resin (EpoFix Resin, Struers, Denmark) was poured into the mold, and the specimens were kept in a humid chamber. After 24 hours, the metal bar was removed and the wax was removed from the coronal surface of the root by the silicon-polishing disc (1200 grit, Struers, Denmark) under water coolant. The specimens were rechecked under 20× magnification for the presence of any dentin crack. The post space preparation was finalized by exact removal of 10 mm gutta-percha from the canal using 1.5-mm-diameter post preparation bur (Rebilda, Voco, Cuxhaven, Germany) at 800-rpm speed. Afterward, the canal was irrigated with distilled water and dried with paper points (Dentsply Maillefer, Ballaigues, Switzerland).

Teeth were randomly divided into two groups (n = 12). Two different FRCs as single cone FRC (Rebilda Post system, Black-coded, 1.5 mm diameter, Rebilda, Voco, Cuxhaven, Germany) and bundled FRC (Rebilda Post GT, 12 single fibers, Black-coded, 1.4 mm diameter, Rebilda, Voco, Cuxhaven, Germany) were luted to the prepared canals according to manufacturer’s instruction. Ceramic Bond (Voco, Cuxhaven, Germany) was applied to the posts, and the posts were left to dry for 60 seconds. Futurabond DC SingleDose (Voco, Cuxhaven, Germany) was applied to the prepared root canal and the coronal surface of the root, excess was removed by paper point and then air-dried. Dual-cure resin-based luting cement (Rebilda DC, Voco, Cuxhaven, Germany) was filled in the canal with caution to avoid air entrapment. The resin luting cement was applied over the post surface and inserted into the canal. Excess cement was removed and the tooth was light-cured for 40 seconds. An almost 4-mm high resin core was built up (Rebilda DC, Voco, Cuxhaven, Germany) and light-cured for 40 seconds. In bundled FRC group, the same procedure was repeated, only the fibers were dispersed before light curing. The length of the FRCs in both groups was adjusted after light curing to the resin core build-up level. Specimens were kept in the humid environment at 37°C for one day.

To examine the tooth fracture resistance, a compressive static load at a 135° angel to the axis of the tooth was applied in the middle of core build-up using a universal testing machine (Instron, Canton, MA, USA). The crosshead speed was set at 1 mm/min. The fracture load was recorded in Newton. The failure type after rupture was identified by visual inspection as A) Core fracture only, B) Root fracture – with or without core fracture, and C) Post fracture – with or without core or root fracture. Tooth fracture resistance data were analyzed at the significance level of 0.05 using student t-test (Stata 11.0, College Station, TX, USA). The fracture mode data were analyzed using Mann-Whitney U test. A sectioned sample in the bundled FRC group after polishing was examined under stereomicroscope for structural assessment.

## Results

**Figure 2:**
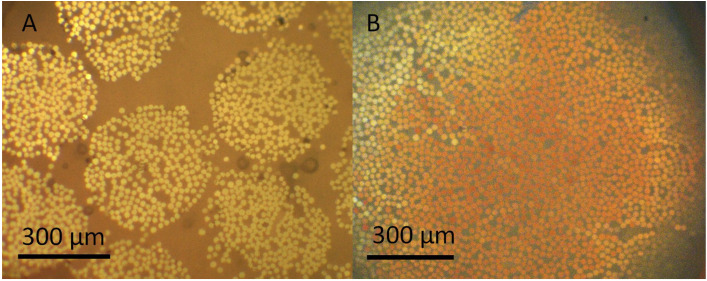
A representative bonded bundle FRC (A) and single FRC (B) to root dentin under stereomicroscope at 25× exhibiting smaller multiple bundles impregnated within a resinous matrix.

Table 1 is presenting the fracture resistance of teeth received single cone FRCs versus bundled FRCs. One specimen in single cone FRCs with 207 Newton and one specimen in bundled FRC with 270 Newton were discarded as outliers after the statistical Grubbs test and were not included in the final statistical analysis. The statistical analysis revealed a non-significant difference in fracture resistance between the two groups (*p* = 0.38). The frequency of fracture types has been summarized in Table 1. The dominant fracture type in the two groups was root fracture (n single FRC=8, n bundled FRC=10) with no significant difference between the groups. The sectioned sample revealed that each bundle in the bundled FRC group and single FRC have been composed of smaller multiple bundles impregnated within a resinous matrix (Fig. 2).

**Table 1:**
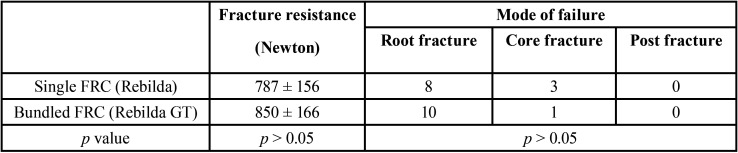
The mean and standard deviations of fracture resistance of teeth received single FRCs and bundle FRCs. The most dominant failure type in both groups was root fracture. Note that one sample in each group was the outlier and discarded from the analysis.

## Discussion

In this study, we evaluated the fracture resistance and failure mode of endodontically treated maxillary incisors treated with bundled FRC or single FRC. Our observations revealed that there was not a statistically significant difference in fracture resistance and failure mode between the two groups. The null hypothesis is therefore accepted.

Restoration of a severely destructed tooth often necessitates insertion of an intracanal post to achieve optimal retention for the restoration. In endodontically treated premolars, with < 2 mm residual coronal dentin walls, placement of a glass-fiber post significantly increases the fracture resistance of the tooth (8). A study has shown that placement of multi posts in a single canal causes a significant decline of stress levels into the residual dentin and consequently decreases the root fracture risk (16). However, the intracanal post placement should be performed without excessive sacrifice of radicular dentin (17) to save existing root structure (3,18) and minimize tooth loss risk due to radicular fractures (19).

In our study, we ensured a correct angel alignment between the post and loading direction by a perpendicular embedding of the specimen using a dental survivor, since a correct and constant oblique loading angulation may create more variation in the results. To our knowledge, this has not been done in other studies. We prepared an identical post space dimension in terms of diameter (1.5 mm) and length (10 mm) in both groups. Single cone FRCs require a certain diameter to exert optimal physical properties and resist fracture under functional and parafunctional loads (5). In contrast, the placement of bundled FRCs is controllable by the number of inserted bundles. Application of bundled FRCs in canals with irregularities e.g. oval or curved canals might most likely be beneficial for better adaptation and custom-made intracanal fiberglass post. This brings a conservative advantageous approach by maintaining more root dentin structure. However, the number of placed bundled FRC should provide sufficient retention of core build-up. The spread of bundles in the coronal structure may also further support better bonding and improved core build-up retention. These are very relevant speculations, which further studies could elucidate.

Our findings are in agreement with Kul *et al*. that showed a non-significant difference in fracture resistance of endodontically treated teeth received single FRC or bundled FRC restored with lithium disilicate crowns. Similarly, they also reported root fracture as the dominant failure type (20). In contrast, Hegde and Arora showed that bundled FRCs (Rebilda post GT system) exhibited maximum fracture resistance compared to elastic FRC post (EverStick fiber post), prefabricated post, and resin-composite build-up without an intracanal post (21). Methodological variations may explain the discrepancies. Note that Hegde and Arora (21). has not disclosed the type and brand of the used prefabricated post, since different post systems can influence the findings.

In our study, we did not standardize the form and height of the core build-up structure; however, the loading point in all specimens was in the middle of core build-up. This most likely caused a similar load induction in the samples. Further studies investigating the height of core build-up, ferrule effect, fracture testing under dynamic loading, and implementation of thermocycling are relevant.

We observed three and one core fracture in single FRC and bundled FRC, respectively. The most dominant failure type following fracture testing was catastrophic root fracture in both groups. The load direction to specimen axis in this study was set to 135 degrees reflecting the positions, contacts, and loading characteristics of upper anterior incisors in Class I occlusion. Oblique load causes a worst-case scenario for fracture resistance testing of endodontically treated teeth (22). Thereby, substantial stress is expected to be induced at the cervical area (23), in absence of any crown and ferrule (20), and convey to root structure causing heavy forces on the post/luting agent/radicular dentin complex causing root fracture. Absence of ferrule regardless of intracanal post type results in root fracture (20).

## Conclusions

Within the limitation of this study, the fracture resistance of endodontically treated maxillary incisors received single FRC or bundled FRC was not significantly different.
